# Potential and limits of Fe-SMA for asphalt crack self-repair: a critical review on thermal FIT & active reinforcement

**DOI:** 10.1039/d6ra06441h

**Published:** 2026-10-26

**Authors:** Michel Muanda Ntoto

**Affiliations:** a School of Materials Science and Engineering, Chang'an University Xi'an 710061 China mchlmuanda@gmail.com

## Abstract

Over the years, studies have shown that fatigue cracking, caused by repeated traffic loads and daily or seasonal thermal cycles, constitutes the most dominant form of degradation in bituminous pavements. To repair it, current repair practices—whether sealing, patching, or thin overlay layers—prove to be passive in nature and rarely last more than one to three years. In parallel, iron–manganese–silicon (Fe–Mn–Si) shape memory alloys (SMAs) have recently been used in civil engineering as an active alternative in concretes and bridges, generating a recovery stress (*σ*_rec_ ≈ 200–400 MPa) upon heating that could, in principle, close a crack from within. This experience inspires the idea of transposing the technique to bituminous pavements. However, to date, any quantitative demonstration of this behavior comes from concrete or steel elements, with no studies on an actual bituminous mixture. This review examines whether the Fe–Mn–Si concept, validated in these other matrices, can reasonably be transposed to pavement maintenance, by confronting SMA thermomechanics against bitumen rheology on three fronts: thermal compatibility, stress transfer through a viscoelastic medium, and practical feasibility, using only data published in viable journals; no new experiments were conducted. This critical analysis revealed three problems: (1) Fe–Mn–Si activation requires 160–300 °C, whereas conventional bitumen degrades irreversibly above about 120 °C, and even a tightly localized Joule or induction pulse brings bitumen within 1 mm of the alloy past this threshold in less than ten seconds, so that the mismatch is effectively unavoidable with current alloys. (2) Once activation is achieved, viscoelastic relaxation acts against it: a recovery stress starting near 200 MPa can drop below 50 MPa within a few days at 20 °C while the surrounding mixture fluctuates, long before the crack lips have a real chance to meet. (3) And where recovery stress does persist, the SMA–bitumen bond is not strong enough to transmit it. The interfacial shear stress, equal to or less than 0.5 MPa, implies anchorage lengths on the order of 150 mm per millimeter of wire diameter, far beyond the typical 10–50 mm of dispersed fibers. The figures of 80–90% crack closure and 50–70% fatigue life extension so often cited in this field come from studies on concrete and are not transferable to asphalt mixtures; no validation on bituminous mixtures of SMA-driven self-healing exists in the published literature. Three priorities follow from this: prepare iron-based alloys with a final activation temperature *A*_f_ below 100 °C and a *σ*_rec_ above 200 MPa; develop heating protocols fast enough to activate the alloy in well under one second; and dedicated characterization of the SMA–bitumen interface, which has never been directly measured. Pass/fail criteria are proposed for a 2030–2032 checkpoint. Concluding that a pathway is not currently viable is not a negative result—it is, in itself, a very useful result.

## Abstract 2

Over the years, studies have shown that fatigue cracking, caused by repeated traffic loads and daily or seasonal thermal cycles, constitutes the most dominant form of degradation in bituminous pavements.

## Graphical abstract

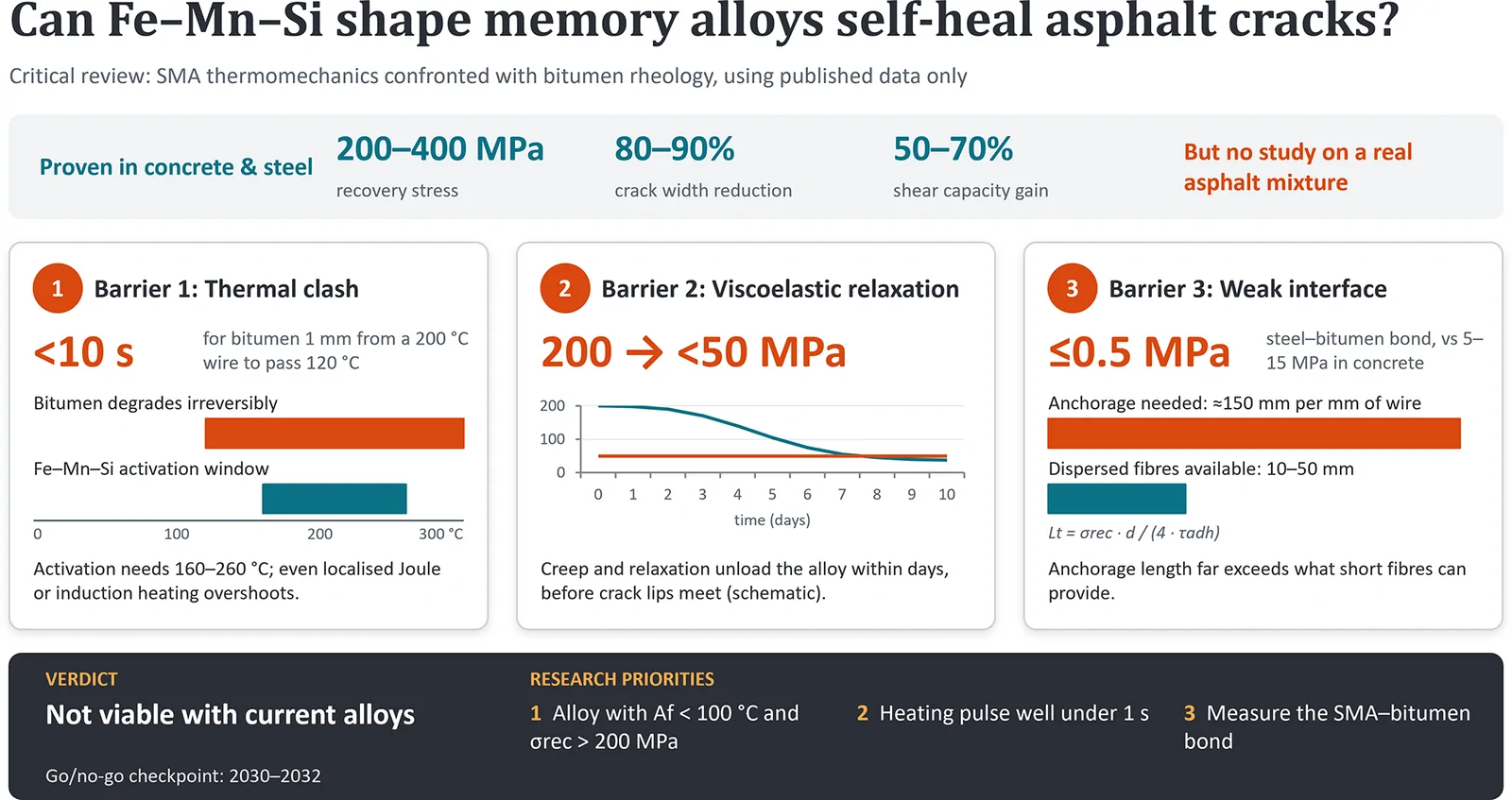

## 1 Introduction

Road networks rank among the largest physical assets held by any country, and keeping them in service is not inexpensive. From EAPA (2022), bituminous mixtures cover more than 90% of the paved road surface in Europe according to Eurostat figures published in 2022, and their maintenance costs several billion euros per year.^69^ Among the anomalies, cracking is the most common and above all the most costly deterioration that these surfaces undergo, and it results from fatigue due to traffic, thermal cycles, and binder aging acting together rather than in isolation.^1,31^

When a microfissure smaller than 100 µm is left to itself, it does not remain in that state for long: water, de-icing salts, and debris infiltrate it, loosen the surrounding aggregates, generate potholes, and reduce the remaining service life of the pavement.^61,70^ The standard toolkit for dealing with this: sealing, thin overlays, localized patching, geosynthetic grids, but all these techniques share the same underlying weakness. Adhesion degrades over time, service life rarely exceeds one to three years on heavily trafficked roads, traffic is disrupted during repair, and none of these methods can do anything once the crack cyclically reopens under load; they are, fundamentally, passive.^47^

In parallel, shape memory alloys (SMAs), which belong to the family of “smart materials”, are attracting growing attention in civil engineering, particularly to overcome the limitations observed in concrete and steel structures.^38,39^ Their characteristic behavior is based on a reversible, diffusionless martensitic transformation, enabling a switch between a high-temperature austenitic phase and a low-temperature martensitic phase,^1^ as illustrated in Fig. 1. Upon heating—whether applied in the superelastic regime or after mechanical deformation—the alloy tends to recover its original geometry; when this recovery is constrained, it generates a significant recovery stress. In practice, two distinct operating modes are exploited: the simple shape memory effect and superelasticity.

**Fig. 1:**
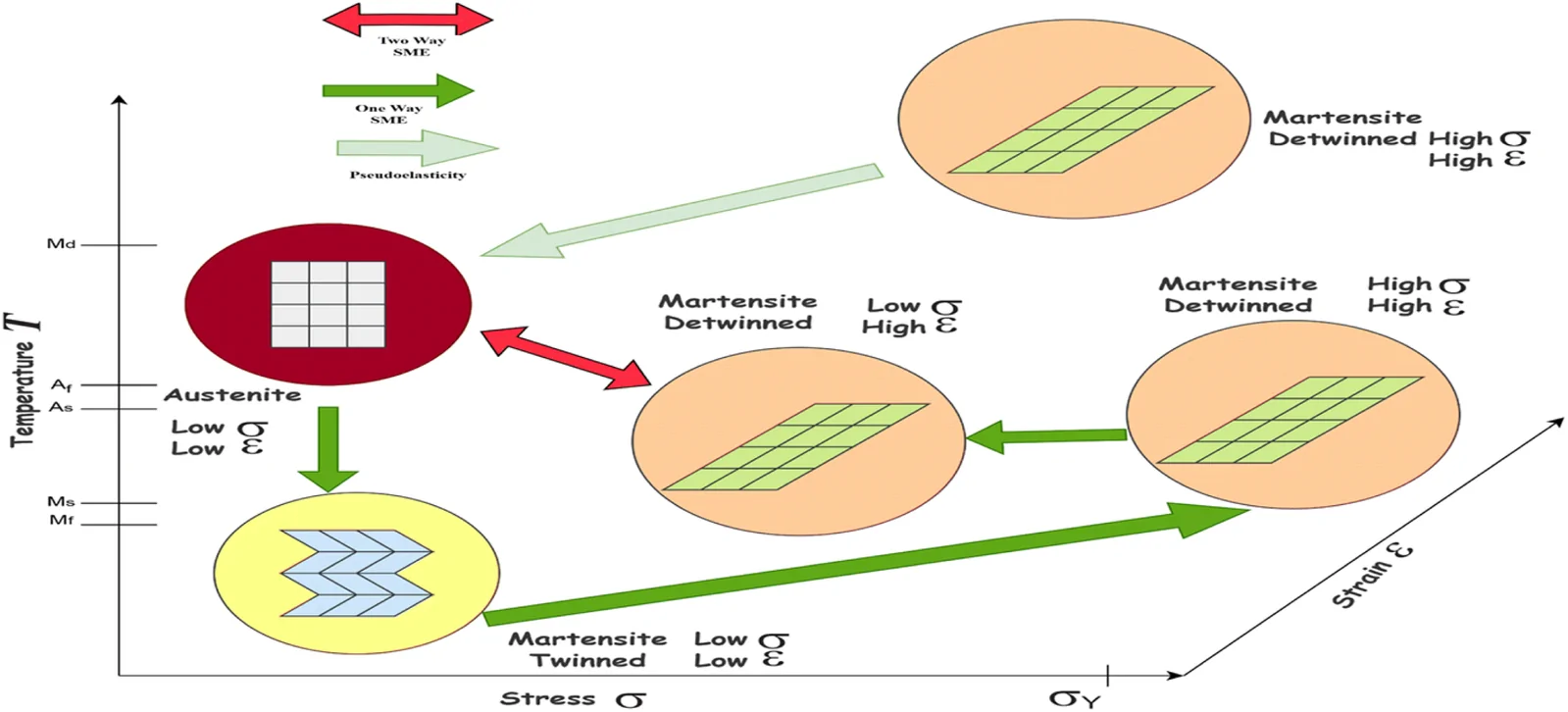
Phase transformation behaviour of an SMA (adapted from ref. 1 with permission from the Royal Society of Chemistry, Hossain *et al.*, Shape memory alloys in modern engineering: progress, problems, and prospects, *RSC Adv.*, 2025, copyright 2025. The original concept was adapted from J. Mohd, *et al.*, *Mater. Des.*,^33^ 2014, **56**, 1078–1113).

Among the SMA family, iron-based Fe–Mn–Si alloys stand out for infrastructure applications because they offer an acceptable balance between cost (approximately 2 to 5 times that of structural steel), weldability, corrosion resistance, and mechanical performance. Recovery stresses of 200–400 MPa are achievable for pre-deformations of 2–6%, with the final austenitization temperature *A*_f_ generally lying between 120 °C and 200 °C.^17,19^ The evidence base for this behavior is now substantial in concrete and steel: embedded Fe-SMA bars activated by Joule heating provide 200–250 MPa of prestress;^4,16^ surface-mounted strips in near-surface mounted,^20^ configurations reduce crack width by 80–90% once heated;^3,18^ and Fe-SMA stirrups increase shear capacity by 50–70%.^40,55^

These results, showed in Fig. 4, have motivated the idea of transposing Fe-SMA technology to bituminous pavements to mechanically close cracks through active self-healing, and to provide adaptive reinforcement that limits crack propagation and compensates for rutting. But a striking observation is this: a careful reading of the literature reveals a gap easily missed upon first glance—no published study demonstrates SMA-assisted self-healing within an actual bituminous mixture under traffic. All performance figures in circulation, without exception, come from concrete or steel, which are matrices different from bitumen.

The critical analysis carried out on the rheological properties of these matrices (concrete and bitumen) allows the identification of four characteristic differences distinguishing bituminous mixtures from concrete.^62,64^ These particularities receive little cross-treatment within existing scientific journals, even though they constitute the predominant criteria governing the transfer phenomenon:^11,62–64,67^

• Thermal sensitivity: bitumen is a viscoelastic thermoplastic that loses most of its stiffness above 50–80 °C, begins to oxidize and harden irreversibly beyond 120 °C, and degrades within a few minutes when it reaches 160–200 °C.^5,22,24^

• Viscoelastic creep: sustained loading produces irreversible deformation in the mixture; a contraction of the SMA intended to close a crack may simply be absorbed by matrix creep before the crack lips actually meet.

• Interfacial adhesion: the steel–bitumen bond stress peaks near 0.5 MPa, compared to 5–15 MPa for steel-concrete; the figures of “bond retention exceeding 80%” common in the SMA literature were measured on concrete or mortar, not on bitumen.

• Environmental aggressiveness: pavements undergo freeze–thaw cycles, de-icing salts, UV exposure, contact with hydrocarbons, and high shear; no study (to our knowledge) has examined the durability of Fe-SMA in contact with a bituminous mixture.

In light of the observations established in the preceding paragraphs, this entire review is guided by a central research question: does the thermal activation of the Fe–Mn–Si alloy enable crack healing in bituminous materials, while preserving the integrity of the bituminous binder?

Three barriers are examined in turn in this review: the temperature ceiling for activation, stress transfer at the SMA–bitumen interface, and the minimum performance that a viable system would require, constructing a quantitative evaluation framework from existing data alone, without new experiments. The scope is deliberately restricted to Fe–Mn–Si alloys in conventional bituminous binders and their thermomechanics, activation, adhesion, and durability; economic analysis and field logistics are excluded.

## 2 Methodology

### 2.1 Justification

We conducted a critical narrative review aimed at testing, through rheological confrontation, whether the transposition of Fe–Mn–Si SMA technology, which has excelled in concrete, could physically and rheologically hold up in bituminous mixtures. This critical review did not aggregate the results from a set of comparable intervention studies, which, as noted in Section 1, do not exist, since no controlled experiment on SMA-reinforced bituminous mixtures under traffic has been published to date. The reporting follows the spirit of the SANRA criteria (justification of the topic's importance, transparent description of the search, solid references, coherent argumentation), adapted here to a question of technical feasibility rather than clinical applicability.^68^

### 2.2 Search strategy

To gather relevant information, we consulted various academic sources, namely: Scopus, Web of Science, and Google Scholar, supplemented by selective searches in *Construction and Building Materials*, *Journal of Materials & Design*, *Journal of Cleaner Production*, *Smart Materials and Structures, Case Studies in Construction Materials*, and other relevant journals where this topic is generally published. Two search chains were executed in parallel. Chain A targeted direct evidence: (‘shape memory alloy’ OR ‘SMA’ OR ‘Fe–Mn–Si’) AND (‘asphalt’ OR ‘bituminous’ OR ‘pavement’ OR ‘road’). Chain B targeted the concrete/steel literature upon which claims of transferability typically rest, to allow for a direct and grounded comparison: (‘iron-based SMA’ OR ‘Fe-SMA’) AND (‘concrete’ OR ‘reinforcement’ OR ‘crack closure’ OR ‘prestress’).

The coverage spans from 2010 to 2026, with the search itself conducted between April and July 2026, without language restrictions beyond English. The reference lists of the most relevant retrieved reviews were manually checked to identify additional primary studies, and forward citation tracking was applied to the most central articles for the transferability argument. Chain A returned very few records, confirming in itself the gap reported in Section 1, while Chain B returned a considerably larger and more mature body of work; this asymmetry is treated as a methodological finding in itself and is reported quantitatively in Table 2. We made use of DeepSeek and Claude, two AI-assisted language models, during the final preparation of this manuscript for language editing, translation refinement, stylistic polishing, and the development of a few figures. Each proposition generated by these models was critically examined, revised, and validated by us, who retain full responsibility for the scientific content and the integrity of the work.

### 2.3 Eligibility criteria

The bibliographic base that informed our research was not chosen at random, but on the basis of strict and rigorous criteria. A study was retained if it reported original, quantitative, peer-reviewed data on at least one of the following: (i) SMA transformation temperatures (*M*_s_, *M*_f_, *A*_s_, *A*_f_) or recovery stress under constrained heating; (ii) SMA bond strength or interfacial shear resistance against a host matrix—concrete, mortar, or bitumen; (iii) temperature-dependent rheological response of bitumen or asphalt mixtures (stiffness, viscosity, relaxation); or (iv) crack closure, prestressing, or fatigue life outcomes attributable to an embedded or near-surface-mounted SMA element. Conference proceedings and unpublished theses were excluded. Purely theoretical or finite-element studies without experimental support were retained but flagged as “modelling only”, so that the reader can distinguish demonstrated behavior from simulated behavior. NiTi- and Cu-based SMAs were retained only for comparative context in Section 3.1 and excluded from the core feasibility analysis in Sections 4 to 6, which is confined to Fe–Mn–Si for the cost and weldability reasons stated. Because this is a narrative rather than systematic review, a formal PRISMA flow reporting was not applied—a deliberate choice rather than an oversight, and acknowledged as a limitation in Section 2.6.

### 2.4 Critical appraisal of included studies

No validated risk-of-bias assessment instrument exists for SMA-civil engineering material studies. A lightweight appraisal grid was therefore applied uniformly to each quantitative value cited for a numerical threshold (*σ*_rec_, *A*_f_, *τ*_adh_ in Tables 1–4), checking: (1) whether the specimen scale and geometry are indicated; (2) whether the host matrix—concrete, mortar, steel, or bitumen—is explicit, *i.e.*, whether the value is used in its native context or extrapolated; (3) whether the measurement method (DSC, restrained tensile test, pull-out test, DSR) is reported; and (4) whether the figure is a single measurement or a range with replicability spread. Values that fail criterion (2)—figures derived from concrete or steel invoked for a bituminous context—are precisely the object of this review's critique and are flagged explicitly wherever they appear in Sections 4 to 6, rather than being silently integrated as if directly applicable.

### 2.5 Data extraction and confrontation framework

For each included study, we collected information regarding alloy composition, transformation temperatures, recovery stress and its saturation strain, bond strength or interfacial shear resistance, host matrix identity, test temperature, specimen scale, and outcome (crack width reduction, fatigue life gain, *etc.*). No quantitative pooling was attempted, no meta-analysis, no forest plot, because the underlying studies differ too greatly in alloy quality, matrix, geometry, and loading regime to support it. Instead, our analysis proceeded by explicit confrontation: for each of the three assessment axes stated in Section 1 (thermal compatibility; mechanical stress transfer in a viscoelastic medium; practical feasibility), the SMA-side figure from the metallurgical literature is placed directly alongside the corresponding bitumen-side constraint from the asphalt literature, and the gap between them is quantified wherever data permit—as in the transfer length calculation of eqn (6.2) or the thermal thresholds in Table 4. When a specific bitumen–SMA value could not be found, the closest available substitute (*e.g.*, steel–bitumen bond stress^3,4,7^) is used and explicitly labeled as such rather than presented as a direct measurement. This confrontation logic—rather than summarizing each article sequentially—is what distinguishes our study here from a conventional literature review: the unit of analysis is the physico-rheological compatibility gap between two bodies of literature that rarely communicate with each other, and not the conclusions of any single study.^8,9^ When this confrontation is expressed in quantitative terms (the thermal diffusivity estimate in eqn (4.2)), the relaxation representation in eqn (5.1), the transfer length calculation in eqn (6.1) and (6.2), all that is being done is to apply established, back-of-the-envelope physical relationships to parameter ranges already reported in the cited literature, to check whether the SMA-side and bitumen-side values, independently published, are actually consistent with each other. No new measurements, specimens, or datasets have resulted from this study; every datum presented here refers back to a published study, and we have performed no experiments. Our contribution has been limited to a critical qualitative analysis.

### 2.6 Limitations of the review methodology

Acknowledging that no human work is perfect, our study was no exception. We are aware that it has certain limitations, notably:

• Single-reviewer process: a single author conducted all searches, screening, and appraisal; without a second independent reviewer, inter-rater reliability could not be verified, and selection bias cannot be entirely ruled out, notwithstanding the explicit criteria in Section 2.3.

• Absence of formal PRISMA reporting: record counts at each screening stage were not tabulated (Section 2.3).

• Grey literature excluded: manufacturers' technical data sheets, patents, and non-peer-reviewed technical reports were not searched, which may under-represent proprietary work on low-*A*_f_ alloys not yet published—relevant to Priority 1 in Section 8.

• Publication bias: the Fe-SMA-in-concrete/steel literature tends toward positive results (crack closure, prestress gains), while null or negative results on SMA-matrix interfaces are less likely to be published—if present, this makes the transferability gap identified here a conservative estimate.

None of these limitations compromise the central conclusion of this review, which rests on well-established thermal and rheological data independently reproduced for SMAs and for bitumen taken separately. However, this does bound the precision of the quantitative thresholds in Tables 4 and 8, which should be read as order-of-magnitude technical targets rather than validated design values. Fig. 2 summarizes the overall methodology.

**Fig. 2:**
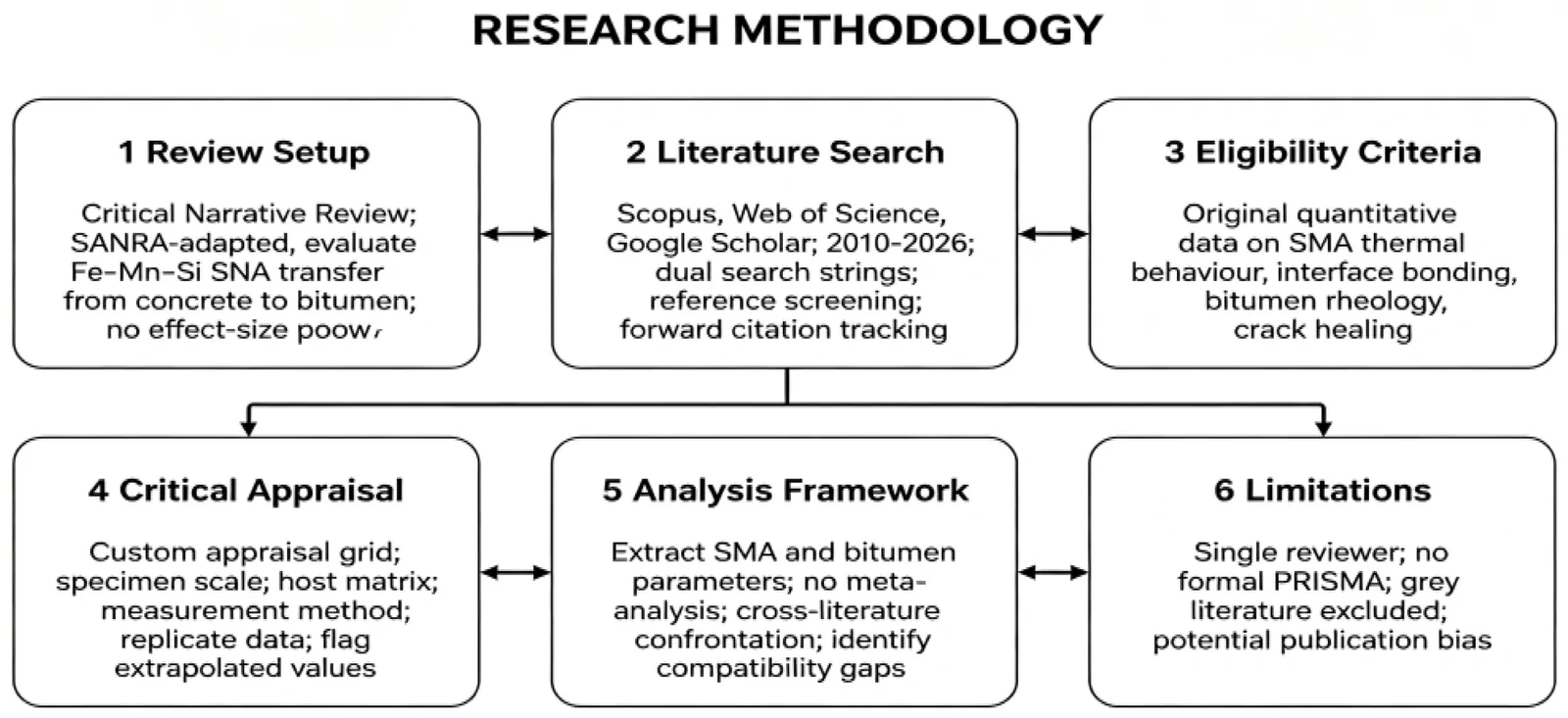
Research methodology.

## 3 Selective background on ferrous shape memory alloys (Fe–Mn–Si)

### 3.1 Scope and justification of choice

Shape memory alloys are metals that recover a previously imposed geometry after plastic deformation, triggered mainly by heat and, in some regimes, by mechanical load alone. The family spans NiTi, copper-based systems (Cu–Al–Ni, Cu–Zn–Al) and Fe–Mn–Si.^34,35^ NiTi delivers the largest recoverable strain (6–8%) and the highest recovery stress^36^ (up to 500 MPa) but costs 10–20 times more than steel and is difficult to weld;^17,19,41^ copper-based alloys are brittle, thermally unstable, and prone to intergranular corrosion.^13^ This review therefore concentrates on Fe–Mn–Si alone, which offers the most workable balance of performance, cost (2–5× steel), weldability and corrosion resistance for road maintenance purposes.^17,33^

### 3.2 Martensitic transformation and characteristic temperatures

The rheology of Fe–Mn–Si is governed by a reversible, diffusionless transformation between FCC austenite (*γ*, stable at high temperature) and HCP martensite (*ε*, stable at low temperature).^33^ Four characteristic temperatures, obtained by DSC or mechanical testing, describe the cycle: *M*_s_ and *M*_f_ (martensite start/finish) and *A*_s_ and *A*_f_ (austenite start/finish), as illustrated in Fig. 1. Above *A*_f_, the alloy is fully austenitic and produces its maximum recovery stress. For the Fe-17Mn-5Si-10Cr-5Ni grade commonly used in civil engineering, as revealed in the studies cited here,^16,17^ its *M*_s_ is near 100–150 °C and *A*_f_ near 120–200 °C, with a thermal hysteresis (*A*_f_ − *M*_s_) of about 30–60 °C. However, Fig. 3 makes visible the underlying conflict: full recovery stress requires heating above about 165 °C, while pavement bitumen begins to degrade irreversibly above 80–120 °C.^6^ Resolving this discrepancy, examined in depth in Section 4, is a prerequisite for any field application.

**Fig. 3:**
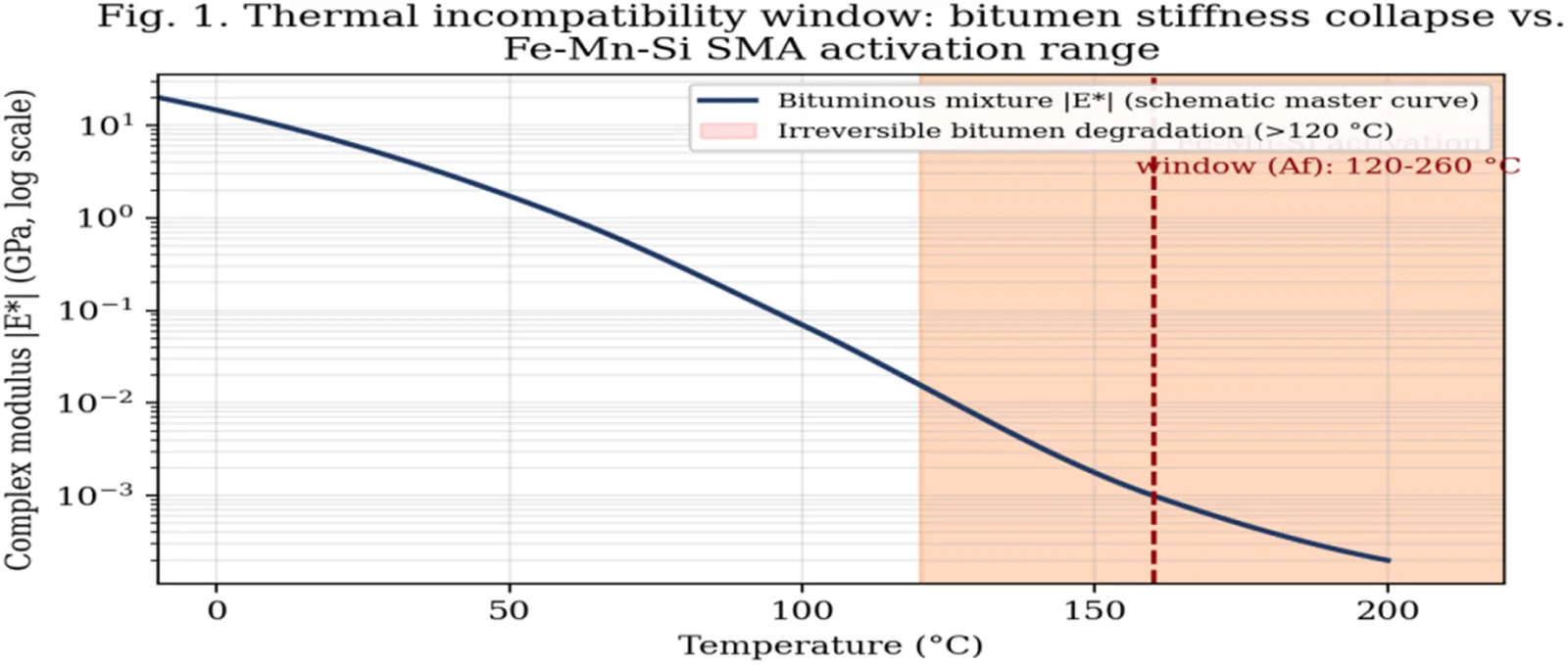
Thermal incompatibility window: schematic bituminous-mixture stiffness master curve (Table 2) against the Fe–Mn–Si activation range (*A*_f_ ≈ 120–260 °C) and the onset of irreversible bitumen degradation (>120 °C).

### 3.3 One-way shape memory effect and recovery stress

Fig. 1 illustrates the phenomenon of the one-way shape memory effect and the recovery stress of AMF. This simple effect, expressing the “intelligence” of these metals, occurs in three steps:^2,33^ pre-deformation below *M*_f_ (reorientation/detwinning of martensite); integration into the structure while still deformed; and heating beyond *A*_f_, which drives the *ε* → *γ* transformation and, if the alloy is constrained, generates a recovery stress. Up to a saturation strain *ε*_max_, *σ*_rec_ increases almost linearly with the pre-strain *ε*_pre_:^17^

3.1 *σ*_rec_ = *σ*_max_(*ε*_pre_) − ESMA × (*ε*_pre_ − *ε*_rec_)

The literature indicates that ESMA for Fe–Mn–Si is approximately 50–70 GPa, and typical compositions saturate around *ε*_max_ ≈ 4–6%, yielding *σ*_rec_ in the range of 200–400 MPa.^16,19^

Given that the strain range of bituminous mixtures (0.1–1%) lies within this linear region, recovery could theoretically be effective. However, this strain-amplitude compatibility does not directly guarantee macroscopic pavement recovery, since stress transfer between the alloy and the bituminous matrix remains a determining factor. It would be possible provided that the activation itself is achievable. Section 4 will demonstrate this issue with a much more coherent and critically examined analysis.

### 3.4 Mechanical hysteresis and energy dissipation

Mechanical hysteresis is a material's capacity to absorb and dissipate a portion of energy when subjected to repeated loading. Mechanical hysteresis and energy dissipation are essential aspects for evaluating the cyclic behavior of SMAs. The stress–strain loop reflects the energy dissipated during repeated loading. For Fe-SMA, this capacity improves damping, limits crack propagation, and contributes to the durability of reinforced structures. Under cyclic loading in the superelastic domain (*T* slightly above *A*_f_), Fe–Mn–Si traces a hysteresis loop whose enclosed area *W*_diss_ is the energy dissipated per cycle:^21^

3.2 *W*_diss_ = ∮*σ*d*ε*

Loop widths of 50–150 MPa are typical for Fe–Mn–Si, compared to 100–300 MPa for NiTi.^17^ No data exist on the cyclic behavior of Fe–Mn–Si embedded in a bituminous mixture, where matrix creep would substantially modify load transfer.

### 3.5 Superelasticity: limited relevance for bituminous mixtures

Superelasticity is the ability of a material, particularly SMAs, to undergo large reversible strains without permanent damage.^2^ It results from a stress-induced phase transformation. This property enables energy absorption, shape recovery, and innovative engineering applications. Above *A*_f_, Fe–Mn–Si can behave superelastically, fully recovering an imposed strain upon unloading without any heating step.^3^ However, our critical analysis reveals three factors that make this largely less likely for pavements: (1) the mixture must first exceed *A*_f_, which is not guaranteed in cold climates; (2) the critical stress required to trigger the transformation (200–300 MPa) is far higher than typical tensile stresses in a mixture (<5 MPa); (3) matrix creep would in any case mask the superelastic response at the fiber level. Superelasticity is therefore, at best, a secondary effect rather than a primary strengthening mechanism here.

### 3.6 Critical appraisal of the concrete/steel evidence base underpinning transferability claims

Today, iron-based SMAs are widely recognized in civil engineering for their remarkable, even unmatched, performance in crack closure. As mentioned in Section 1, under thermal activation, these smart metals develop recovery stresses on the order of 200 to 250 MPa, generating a prestressing effect that actively tightens cracks and promotes self-repair, which is very important for ensuring matrix durability.^4,16^ Heating the matrix activates this operation and reduces crack widths by 80 to 90%,^3,18^ while simultaneously improving shear strength by 50 to 70%.^55^ These figures, summarized in Fig. 4, are not generic; they illustrate the transformative potential of smart materials for structural rehabilitation, and underpin the claims of transferability of this technology to the bituminous matrix, which struggles to withstand these environmental stresses.^42,43,46,48,53,54,56,58^ However, these promising results come essentially from laboratory studies on concrete/steel. The central question raised by this review is precisely whether these performances will hold true at the scale of real pavements. Fig. 4 present main performance results of Fe-SMA from concrete and steel studies.

**Fig. 4:**
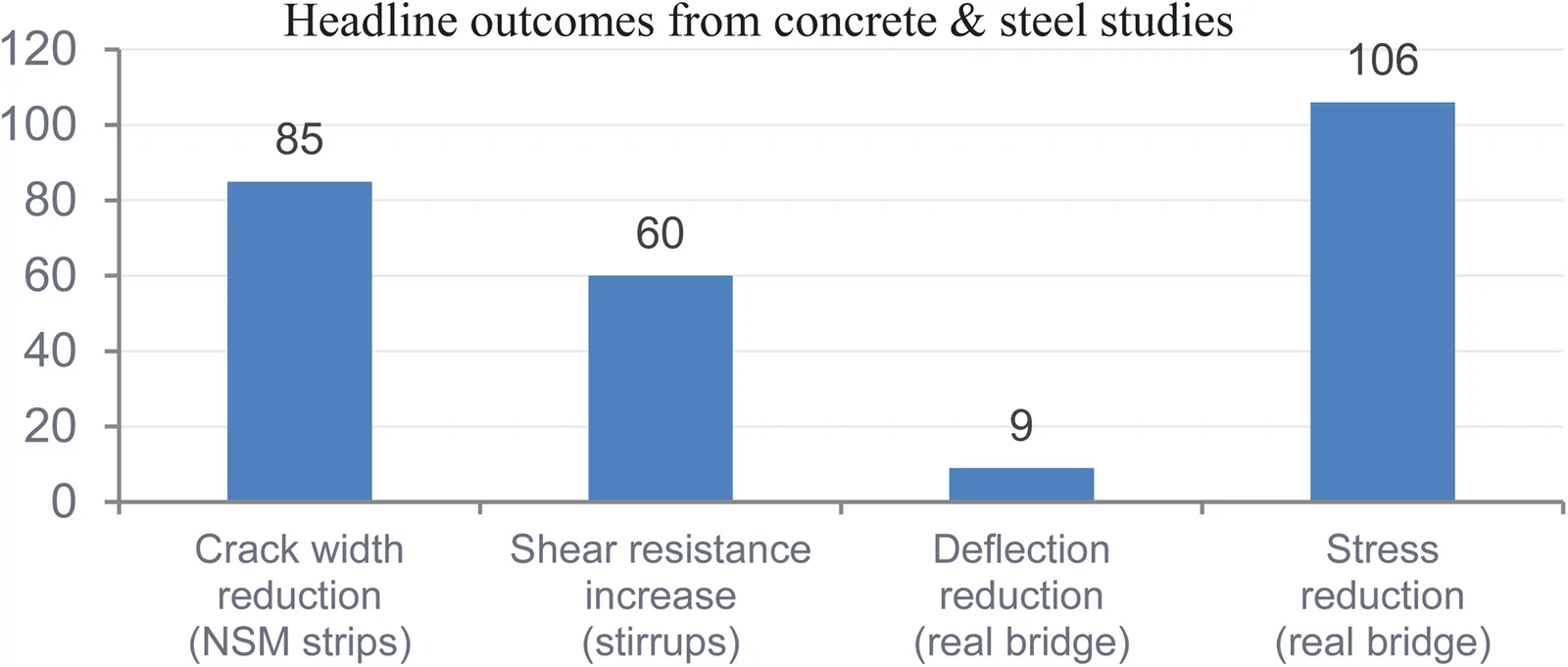
Fe-SMA performance headline outcomes from concrete & steel studies.

Before confronting the data drawn from Fe-SMA performance on concrete/steel with bitumen rheology (Sections 4 to 6), it is appropriate to critique the quality of the data themselves, because the relevance of the transferability idea can only be as sound as the scientific basis on which it rests. A critical examination of the studies that provided the flagship figures attesting to this performance reveals three weaknesses: (1) researchers have largely used the same alloy suppliers and the same testing protocols, for example, the Empa group being among the most prominent;^3,4,16,18,19,44,45,63^ (2) the figures of 80–90% crack closure and 50–70% shear capacity thus look less like an independently reproduced consensus than like results from the same experimental tradition reported many times over, and the recurring nearly identical figures in these articles may simply reflect a shared method and specimen population rather than validation by unrelated groups. Nearly all also come from small-scale specimens, bars, strips, or beams typically a few meters long, tested at controlled temperature over a limited number of cycles, with large-scale validation under real traffic conditions limited to a handful of pilot bridges^37,49^ rather than a broad structural population. (3) Long-term field data beyond a few years remain scarce, even for concrete and steel,^45^ so much of what is cited as “established” Fe-SMA performance is itself short-term laboratory evidence. However, none of these limitations undermine the results obtained on concrete and steel themselves. On the other hand, they show that the data used to extrapolate to bituminous mixtures are more limited and more tightly confined to the laboratory setting than the overly optimistic claims about process transferability suggest. This caveat reinforces the importance of the physical incompatibilities presented subsequently, rather than merely complementing these observations. Table 1 evaluates, property by property, to what extent the characteristics of Fe–Mn–Si match what a bituminous mixture would actually require. Table 1 assesses, property by property, how well Fe–Mn–Si characteristics map onto what a bituminous mixture would actually require.

**Table 1:** Fe–Mn–Si properties against bituminous-mixture requirements^a^

Property	Fe–Mn–Si value	Critical threshold (mixture)	Method	Critical comment
*A*_f_ (°C)	120–200	<80 (service); <120 (peak)	DSC/dilatometry^16,17^	Bitumen degrades above 120 °C—direct activation impossible without matrix damage.
*σ*_rec_ (MPa)	200–400	>1–2 (to overcome bitumen cohesion)	Blocked tension test^19,51^	Asphalt fails near 0.3 MPa; transfer is uncertain given creep and unknown relaxation.
*ε*_max_ (max. recoverable strain)	4–6%	0.1–1% (cracking strain)	Cyclic tension + heating^17^	Cracking occurs well below 4%; the surplus deformation cannot be exploited.
Young's modulus (GPa)	50–70	1–15 (*T*- and frequency-dependent)	Tension, initial slope^19^	Large stiffness mismatch drives stress concentration and premature debonding.
Corrosion resistance	≈Stainless 304	>10 years service life on roads	Salt spray ASTM B117 (ref. 17 and 33)	No data for SMA interfaces in aged bitumen, acids or chlorides.
Relative cost	2–5× steel	<10% extra cost for wide adoption	Market analysis^13,33^	Prohibitive for routine maintenance; viable only for critical zones (<1% of area).

a Fe–Mn–Si alloys (*A*_f_: 120–200 °C; *σ*_rec_: 200–400 MPa; *ε*_max_: 4–6%) exceed bitumen thresholds, which degrades above 120 °C and cracks at 0.1–1%. High stiffness and cost restrict their use to critical zones (<1%).

## 4 Thermal compatibility between Fe–Mn–Si activation and bituminous mixtures

### 4.1 Problem statement

The bituminous matrix is a viscoelastic material, more compliant, capable of dissipating energy and deforming under load. The existing literature on the rheology of the bituminous matrix^6,10,59^ reveals that this material exhibits a strong sensitivity to temperature variations. The gap between concrete or steel and the bituminous matrix stems essentially from their nature, their mechanical behavior, and their degradation mechanisms. Beyond the foregoing, the main obstacle to incorporating Fe–Mn–Si into a bituminous pavement is temperature, because the temperature required for a recovery stress to be fully activated lies between 160–260 °C;^4,16,17^ however, bituminous binders modify and degrade irreversibly above temperatures of about 100–120 °C.^23,59,64^ This thermal conflict is illustrated by Fig. 3. Experience on concrete and steel shows a tolerance to this range without significant damage, which is intolerable for bituminous mixtures that degrade progressively and permanently.

### 4.2 Thermomechanical behaviour of bitumen: master curves

Several studies have been conducted on the thermoplastic viscoelastic behavior of bitumen, demonstrating the critical importance of understanding its rheology. The analysis of this literature reveals a frequent representation of the thermoplastic viscoelastic behavior of bitumen through master curves of the complex modulus |G*| referenced at 20 °C.^26,64^ In Table 2, we present representative stiffness values according to the relevant temperature range.

**Table 2:** Complex modulus of a bituminous mixture as a function of temperature^a^

Temperature	Complex modulus |E*|	Behaviour	Consequence for SMA activation
−10 °C	15–25 GPa	Brittle elastic	Risk of thermal cracking
20 °C	5–10 GPa	Viscoelastic	Reference service condition
60 °C	0.5–2 GPa	Soft, rutting risk	Matrix too soft to transmit SMA stress efficiently
100 °C	<0.1 GPa	Quasi-liquid	SMA contracts in a fluid medium—no crack-closure force transmitted
160–200 °C	Viscosity <1 Pa s	Fluid, non-load-bearing	SMA activation range—bitumen is completely fluid

a The SMA activation window (160–200 °C) coincides with a fully fluid bitumen, so no stress can be transmitted. At service temperatures (−10 to 60 °C), the matrix is either too brittle (thermal cracking) or too soft (rutting). The optimal zone (20 °C) is viscoelastic, but the SMA cannot be thermally activated there. This reveals a fundamental thermo-mechanical incompatibility.

### 4.3 Thermal degradation of bitumen: mechanisms and kinetics

The bituminous matrix is subject to attacks from several environmental factors (rain, cold, heat, traffic, *etc.*). Among these degrading sources, this matrix proves to be much more sensitive to that triggered by thermal variation. When heated above about 80 °C, a degradation process slowly and progressively activates, causing the breaking of molecular bonds and promoting oxidation of the matrix.^29,30^ It is important to note that the rate of this oxidation reaction is not always uniform in all cases. It depends on external factors such as heating temperature, exposure time, and ambient air; the synergy of these agents modifies the flexibility and strength of the matrix. Studies on the description of thermal degradation of bitumen indicate several processes of this degradation:^23,59,64^

• Physical softening (80–120 °C, partially reversible): within this thermal range, a drop in stiffness and a possible disturbance of the colloidal microstructure in the matrix are observed.

• Accelerated oxidation (>100 °C, irreversible): above 100 °C, degradation accelerates and causes the formation of by-products such as carbonyl and sulfoxide compounds.^12^ In this regard, simulation studies on the behavior of the bituminous matrix exposed for a few minutes at 150 °C revealed a degradation level approximately equivalent to several years of natural aging. This reinforces the thesis of the great sensitivity of bitumen to high temperature.^31,32^

• Loss of light fractions (>120–150 °C, irreversible): when a temperature above 150 °C is reached, the bituminous matrix decomposes, aromatic oils and saturates evaporate; consequently, the binders harden and the bitumen-aggregate adhesion weakens greatly.

• VOC/fume emission (>160 °C): chemical–organic theory indicates that organic compounds are mainly composed of a carbon skeleton, and their combustion primarily produces energy, water, and CO_2_. Therefore, when heating of the matrix reaches a temperature above 160 °C, degradation becomes complete, volatile organic compounds escape, raising safety and environmental concerns.

• Carbonization (>200 °C): cracking reactions leave gaseous hydrocarbons and a friable carbonaceous residue.

Other studies on thermal spikes suggest that brief localized exposure at 150 °C for less than two minutes, combined with a rapid cooling technique, could be tolerable, but above this temperature (for example at 200 °C), even for thirty seconds, irreversible local damage has been observed.^66,67^Table 3 synthesize thermal effects on conventional bitumen and their consequences.

**Table 3:** Thermal effects on conventional bitumen (grade 35/50)^a^

Temperature	Critical duration	Dominant effect	Reversibility	Consequence for SMA activation
80–100 °C	>1 h	Softening	Partial	Recoverable stiffness loss on cooling
100–120 °C	>10 min	Mild oxidation	Irreversible	Accelerated ageing, embrittlement
120–160 °C	>1 min	Volatile loss, strong oxidation	Irreversible	Hardening, adhesion loss
160–200 °C	>30 s	Fume emission, onset of cracking	Irreversible	Local matrix destruction
>200 °C	Seconds	Carbonisation	Irreversible	Unacceptable under any scenario

a AMF activates between 160–200 °C, where bitumen is irreversibly destroyed within 30 seconds. Below 120 °C, it ages slowly. The reversible zone (80–100 °C) is too cold for AMF. No safe temperature window exists: a fundamental thermomechanical incompatibility.

### 4.4 Localised activation by Joule effect and induction: principles and limits

For the Fe-SMA to activate its smart properties and self-repair cracks in the matrix, it must be heated. However, it is crucial to always keep in mind the sensitivity of bituminous material to uncontrolled heating. Consequently, heating the entire matrix that hosts the alloy is risky. To address this, two localized heating techniques prove effective: (1) Joule heating: this technique requires an electric current that acts through resistance to produce localized heat in the matrix. It relies on the physical law known as the “Joule–Lenz law”. (2) Induction heating: this technology relies on the physical law known as the “Faraday–Lenz law”. It uses a magnetic field that generates eddy currents to produce heat directly in the material. To complement the above, resistive heating (Joule effect) passes a current directly through the Fe-SMA wire or bar, which dissipates power per unit volume.^3,18^Eqn (4.1) expresses this mathematically:

4.1 *P* = *j*^2^ × *ρ*

where: *j* is the current density and *ρ* ≈ 70–100 µΩ cm for Fe–Mn–Si; applying 10–50 V can reach 200 °C within a few tens of seconds.

These methods are fast and targeted, perfect for spot repairs. But they have their weaknesses: high energy consumption, thermal gradients that are difficult to control, and efficiency that depends heavily on the conductivity of the treated product.^63^Fig. 5 illustrates the technique of heating a bituminous matrix by Joule effect.

**Fig. 5:**
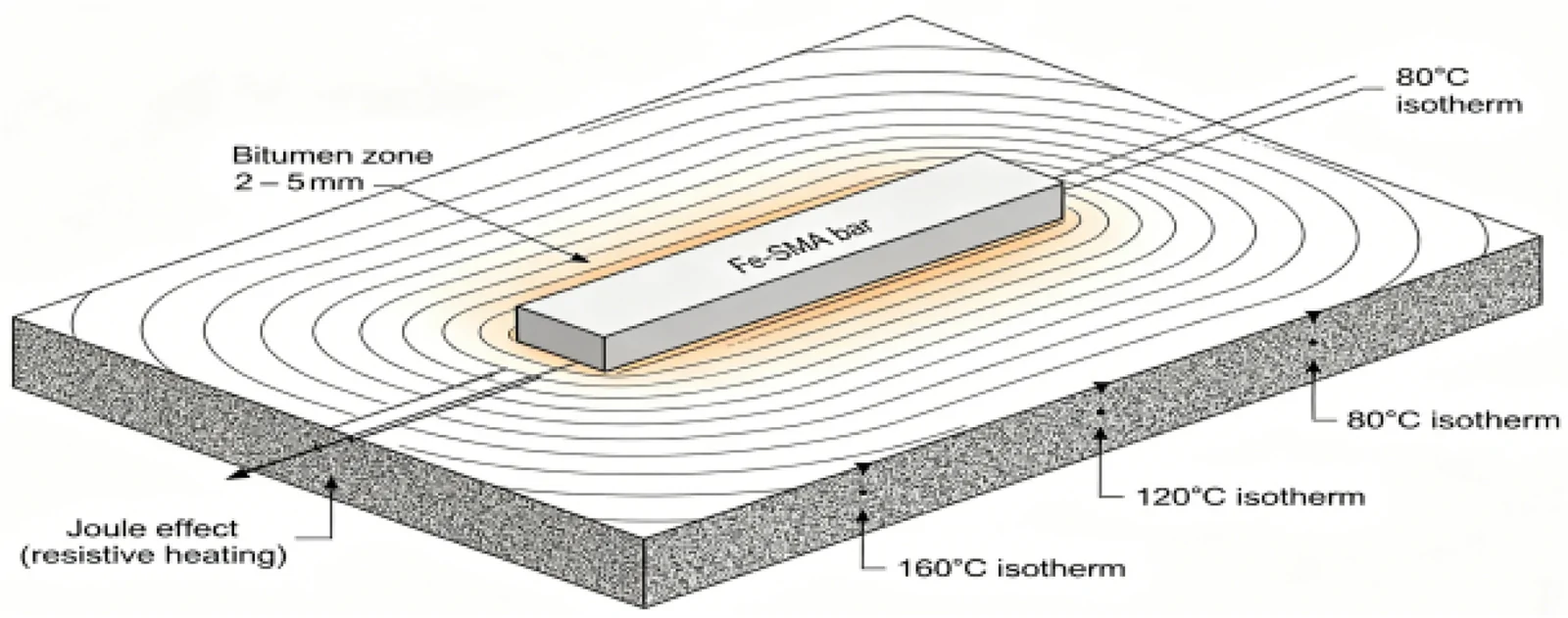
Joule heating of an Fe-SMA bar in a bituminous mixture.

#### 4.4.1 Independent quantitative check: radial heat-diffusion time

The radial thermal time represents the time required for the heat generated by the Fe-SMA to propagate and reach the surrounding bitumen, thereby enabling thermal activation. Infrared thermography test results from study^63^ indicate that a 1 mm Fe-SMA wire at 200 °C in bitumen can raise the matrix temperature beyond 120 °C within 10 seconds; yet at this threshold, the stability of the matrix is severely threatened. To circumvent this, one can use the theory of one-dimensional transient heat conduction. In this framework, for radial diffusion from a thin cylindrical source, the characteristic time *τ*_diff_ to reach a distance *L* follows the Fourier number relationship presented in eqn (4.2):

4.2 *τ*_diff_ ≈ *L*^2^/*α*

where: *α* is the thermal diffusivity of the mixture, reported for dense bituminous mixtures in the range of 3.5 × 10^−7^ to 5.0 × 10^−7^ m^2^ s^−1^, reflecting both the mineral skeleton (*k* ≈ 2–3 W m^−1^ K^−1^) and the much less conductive bituminous mastic (*k* ≈ 0.15–0.20 W m^−1^ K^−1^).^27,65^

For a characteristic distance of *L* = 1 mm, the theoretical model in eqn (4.2) predicts a thermal diffusion time *τ*_diff_ of approximately 2 to 3 seconds. This value is consistent with the experimental observations of about 10 seconds reported in study.^63^ This agreement is important because it shows that the rapid thermal contamination of bitumen around an activated SMA stems from the thermal properties of the bituminous mixture itself, and not from an effect related to the experimental setup. Since *τ*_diff_ scales with *L*^2^, doubling the separation distance to 2 mm only extends the safety window to about 10–12 seconds. However, for complete and effective activation of the reverse transformation of Fe-SMAs, it is imperative to maintain the temperature at *A*_f_ for several minutes. This finding confirms that the layout and spacing of Fe-SMA bars in the bituminous matrix play an essential role in ensuring uniform and efficient thermal activation. Fig. 6 confronts the analytical radial thermal diffusion time (*τ*_diff_ ≈ *L*^2^/*α*) derived from eqn (4.2) with the experimental data point from,^63^ plotted across the diffusivity range of bituminous mixtures.

**Fig. 6:**
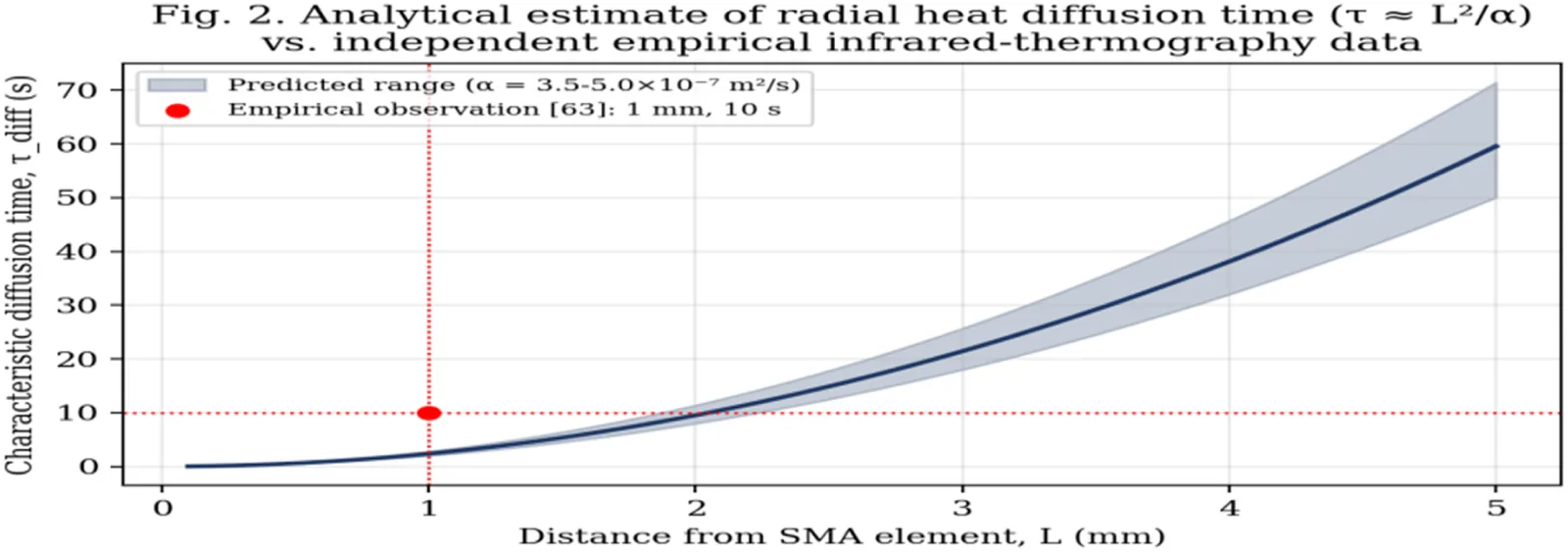
Analytical radial heat-diffusion time (*τ*_diff_ ≈ *L*^2^/*α*) *versus* distance from an activated Fe–Mn–Si element, plotted across the reported bituminous-mixture diffusivity range, against the independent empirical data point from.

### 4.5 Acceptable thermal thresholds: a proposal for bituminous mixtures

The thermal constraints we have just examined point to a remarkably narrow operating window for SMA activation in bituminous mixtures. Table 4 synthesizes these constraints into a set of provisional thermal thresholds for conventional bituminous mixtures (grades 35/50 or 50/70). These values draw on the degradation mechanisms and thermal diffusion time analysis developed in the previous sections.

**Table 4:** Proposed SMA activation thresholds for conventional bituminous mixtures (grade 35/50 or 50/70)^a^

Parameter	Threshold value	Justification
Max. temperature in bitumen core	<100 °C	Avoids rapid volatile loss and severe oxidation^59,64^
Exposure duration at 100–120 °C	<30 s	Limits diffusion and degradation kinetics^66^
Max. temperature at SMA–bitumen interface	<150 °C (peak only)	Even brief exposure above this causes irreversible local damage
Required *σ*_rec_ under these constraints	>200 MPa (target)	Achievable only with low-*A*_f_ Fe–Mn–Si, not yet commercially available

a This table defines the thermal and mechanical thresholds to be met in order to activate a shape memory alloy (SMA) in an asphalt pavement without degrading the matrix. The *σ*_rec_ (recovery stress) is the stress developed by the SMA to close cracks; *A*_f_ (austenite transformation finish temperature) is the minimum activation temperature for the memory effect.

The critical analysis of this table reveals a harsh operational conclusion: with the existing Fe–Mn–Si alloy, whose *A*_f_ value is approximately 120–200 °C, since around this temperature activation without degradation of the bitumen is less feasible once the exposure exceeds a few seconds. For this to be possible, only alloys with *A*_f_ below 100 °C must be used, which could in principle be activated without this compromise; however, to our knowledge, these alloys are not yet commercially available.

## 5 Self-repair mechanisms *via* Fe-SMA *vs.* asphalt viscoelastic behaviour

### 5.1 Problem statement

The self-healing properties of Fe-SMA alloys are based on their shape memory effect, which is activated by thermal input. This means that Fe-SMAs provide the bituminous matrix with the ability to self-heal actively, thanks to their smart characteristic of recovering their original shape and acting as an intelligent reinforcement that exerts an effective crack-closing force. A review of the existing literature on Fe-SMAs reveals four mechanisms by which Fe–Mn–Si could contribute to the self-healing or active reinforcement of the bituminous matrix: active crack closure, thermal stress compensation, rutting reduction, and fatigue life extension. These four mechanisms have been proven valid on concrete and steel, where they have demonstrated the performance shown in Fig. 4; this regularly encourages the motivation to transfer this technology to bituminous mixtures, without taking into account the rheological differences that separate the two matrices.

### 5.2 Viscoelastic properties of bituminous mixtures

Visco-elasticity is the ability of bitumen to behave both as an elastic material and as a viscous material. This ability helps the matrix control deformation, energy propagation, fatigue, and even self-healing under repeated loading. However, this same property sometimes acts to the disadvantage of this new technology and can cause the failure of this transferability. Three properties fundamentally alter the picture of stress transfer compared to concrete:^7,15,26^

• Creep: the bituminous mixture deforms in a delayed and irreversible manner under sustained stress. For a pressure of 1 MPa at 20 °C, creep strain can reach 0.1–0.5% within a few hours.^60^

• Stress relaxation: stress decreases exponentially under constant imposed strain. An initial stress of 1 MPa can drop to 0.2–0.3 MPa within a few hours at 20 °C.

• Non-linear behavior: beyond approximately 0.1–0.5% strain, the mixture enters a non-linear regime with accumulation of permanent deformation.

Note that any mechanism that depends on maintaining compressive stress over time, in particular crack closure and prestressing within the matrix, proves vulnerable to creep and relaxation in a way that has no real equivalent in concrete.^50^

### 5.3 Active crack closure by shape memory effect

#### 5.3.1 Principle and claimed performance

The principle of self-healing of cracks by shape memory effect relies on contraction after thermal activation. This contraction acts as a restoring force that brings the two edges of a crack closer together, enabling the crack to close. Experiments on concrete have shown that a pre-deformed Fe-SMA element embedded in the concrete mixture contracts once heated beyond *A*_f_, generating *σ*_rec_ (200–400 MPa) that brings the crack lips closer together (Fig. 4). This allows an 80–90% reduction in crack width.^3,18^

#### 5.3.2 Critical analysis for bituminous mixtures

For the case of the bituminous matrix, a technological transfer failure appears likely due to three obstacles:^57,61^

• Creep absorbs the recovery stress: the mixture around the SMAF creeps under *σ*_rec_, partially negating the expected crack closure; when creep is rapid, the crack may reopen as soon as heating ceases.

• The residual stress relaxes: relaxation models for bitumen suggest that an initial *σ*_rec_ of 200 MPa would drop below 50 MPa within a few days at 20 °C, although no data yet exist on the relaxation kinetics for real SMAF-mixture pairs.

• The matrix is essentially liquid during activation: reaching *A*_f_ (120–200 °C) heats the surrounding mixture well beyond the softening point, into a quasi-liquid state (viscosity <1 Pa·s), so that the SMAF contracts without transmitting significant stress; stress transfer only becomes possible after cooling, at which point contraction is already complete and the geometry is fixed. Consequently, the net effect of the operation is practically zero. This relaxation can be formalized using a generalized Maxwell representation (Prony series) of the linear viscoelastic relaxation modulus *E*(*t*) of bitumen, the standard formalism behind DSR master curves:

5.1 *E*(*t*) = Σ_*i*_*E*_*i*_ × exp(−*t*/*τ*_*i*_)

where *τ*_*i*_ are the discrete relaxation times and *E*_*i*_ the associated partial moduli. For conventional pavement bitumens 35/50 or 50/70 at 20 °C, relaxation times reconstructed from DSR frequency sweeps range approximately from 10^−2^ to 10^4^ s, from the sub-second scale to several hours.^62,65^

A constrained SMAF element against the mastic therefore never maintains a fixed recovery stress: the surrounding matrix behaves as a distributed network of springs and dashpots that progressively unload under the imposed strain, exactly as in classical stress relaxation under fixed displacement limits (Fig. 6). Since the longest relaxation times of the binder (10^3^–10^4^ s, *i.e.*, tens of minutes to a few hours) already account for most of the modulus decay at 20 °C, and creep at the aggregate-mastic scale adds a slower unloading mechanism on top of what the Prony parameters of the binder alone capture, the reported drop in *σ*_rec_ from about 200 MPa to below 50 MPa within a few days is consistent with, and not merely asserted alongside, the known relaxation spectrum of pavement bitumen.

A critical analysis of the enhanced relaxation of the recovery stress of an SMAF embedded in a viscoelastic bituminous matrix, as presented in Fig. 7, allows us to conclude that with the current Fe–Mn–Si (*A*_f_ > 120 °C), active crack closure is not a feasible mechanism in the bituminous matrix.

**Fig. 7:**
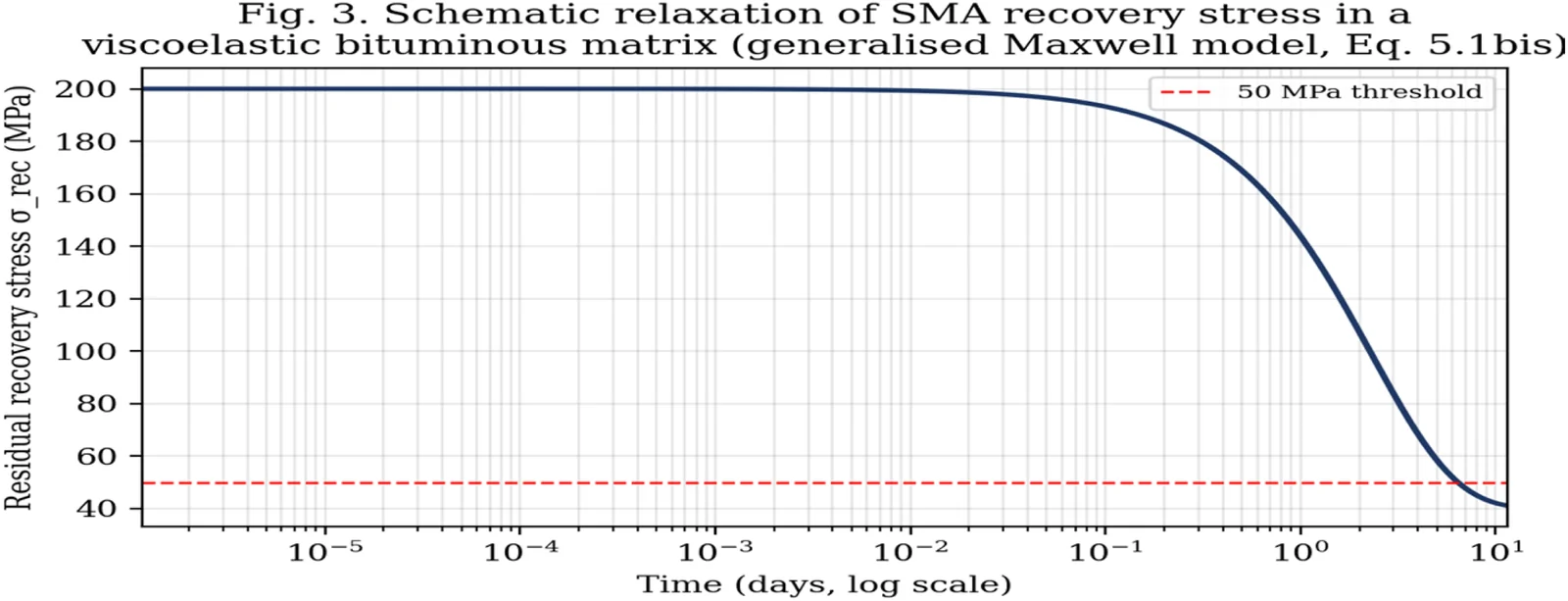
Schematic relaxation of SMA recovery stress embedded in a viscoelastic bituminous matrix, generalised-Maxwell representation (eqn (5.1)) calibrated to the qualitative decay range discussed in the text.

### 5.4 Thermal stress compensation

Fe-SMAs have a temperature-dependent coefficient of thermal expansion *α*SMA(*T*) that could, in principle, compensate for the expansion or contraction of the mixture and reduce residual thermal stress. A proposed model (eqn (5.2)) assumes linear-elastic behavior for both constituents. For bituminous mixtures, this assumption does not hold. Moreover, thermal stresses in an unreinforced mixture are only 0.5–2 MPa for a 30 °C variation,^27^ two to three orders of magnitude below *σ*_rec_ (200–400 MPa), so that the risk is local overstressing rather than compensation; and no study has actually demonstrated the compensation of thermal stresses by SMAs in a bituminous mixture.

5.2 *σ*_therm_ = ∫[*T*_0_ to *T*][ESMA(*T*) × *α*SMA(*T*) − *E*_asphalt_ × *α*_asphalt_]d*T*

### 5.5 Rutting reduction

Rutting is a permanent deformation of the pavement caused by repeated traffic, temperature, and aging. Pre-deformed SMA fibers with an initial shape could, in theory, serve as a solution by exerting a recovery force capable of counteracting rut formation under traffic.^25^ However, three problems compromise this: activation requires *A*_f_ ≈ 120–200 °C, yet pavement surface temperatures in summer barely reach 60–70 °C (up to 80 °C in desert climates), so solar activation is out of reach and, above all, ineffective at generating the vertical recovery force needed to counteract rutting. Moreover, no study examining rutting reduction based on SMAs in a bituminous mixture has been published to date.

### 5.6 Fatigue life extension

Under cyclic traffic loading, hysteretic dissipation in Fe–Mn–Si could in principle reduce the energy available for fatigue crack propagation, sometimes expressed by a modified Basquin law.^1,28^ This is the most defensible of the four mechanisms, but two limitations may remain: dissipation only becomes significant above the martensitic transformation stress threshold of 100–200 MPa, whereas typical traffic-induced stresses in a mixture are 0.5–2 MPa—so no transformation is triggered and the SMAF behaves as an ordinary elastic fiber; and the viscoelastic hysteresis inherent to the mixture (10^4^–10^5^ J m^−3^ per cycle) would overwhelm any dissipative contribution from the SMAF even if it did occur.

5.3 *N*_f_(*σ*,*T*) = *C* × (*σ*/*σ*_ult_(*T*))^*m*(*T*)^

### 5.7 Synthesis of mechanisms

We revisit the four mechanisms discussed earlier: crack closure, balancing of stresses caused by temperature variations, rutting reduction, and extended fatigue service life. We conduct an in-depth analysis of these aspects considering the typical viscoelastic behaviour of bituminous mixtures. Table 5 compares the practicality of each process, activation requirements, and scientific constraints restricting the application of Fe-SMA within bituminous mixes.

**Table 5:** Critical evaluation of SMA self-repair and active-reinforcement mechanisms in bituminous mixtures^a^

Mechanism	Validated on concrete	Transferability to mixture	Main obstacle	Recommendation
Active crack closure	Yes (80–90%)	Very low	Matrix creep + activation temperature too high	Abandon with current Fe–Mn–Si; study *A*_f_ < 80 °C alloys
Thermal stress compensation	None	Zero	Incompatible stress orders of magnitude	Do not present as an expected benefit
Rutting reduction	None	Zero	Solar activation impossible; fibre orientation incompatible	Do not present as an expected benefit
Fatigue life extension	Indirect (steel structures)	Moderate	Transformation stress threshold not reached under traffic	Study stress concentrators (special geometry, small-diameter fibres)

a The table calls for a realistic repositioning of SMA research in asphalt—focusing only active crack closure and fatigue extension show potential in bitumen; both face major obstacles (creep, high *A*_f_, insufficient stress). Thermal compensation and rutting reduction have zero transferability and should not be claimed. Future work needs *A*_f_ < 80 °C alloys.

## 6 Technological obstacles to integrating SMAs into bituminous mixtures

### 6.1 Adhesion and load transfer at the SMA–bitumen interface

The adhesion at the interface between two distinct matrices represents the capacity of their contact area to ensure an effective transfer of stresses. It results from the chemical, physical, and mechanical interactions between the phases. The existing literature reveals that the Fe-SMA-concrete adhesion stress is 5–15 MPa, and surface treatment techniques improve it by an additional 20–30%.^3,19^ In parallel, the peak steel–bitumen adhesion stress is typically 0.2–0.5 MPa at 20 °C and below 0.1 MPa at 60 °C;^3,4,7^ ferrous alloys wet poorly against apolar bitumen (contact angle greater than 90°),^60^ and the interfacial bitumen film lubricates at high temperature and adheres only weakly upon cooling.^62^ This stress can be described by a simple shear force equilibrium calculation. Take the example of a shape memory alloy (SMA) fiber of diameter *d*, embedded over a length *L* within the bituminous mastic. The assembly undergoes an interfacial adhesion shear stress *τ*_adh_, which is assumed to be constant over the entire length *L*. This assumption is both conservative and favorable to the calculations: in reality, the measured adhesion stress gradually decreases with distance from the loaded end of the fiber. By equating the axial force carried by the fiber cross-section with the shear force mobilized over the embedded cylindrical surface, we obtain:

6.1 *σ*_rec_ × (π*d*^2^/4) = *τ*_adh_ × (π*d* × *L*)

Solving for *L* gives the minimum anchorage (transfer) length required to mobilize the full recovery stress:

6.2 *L*_*t*_ = (*σ*_rec_ × *d*)/(4 × *τ*_adh_)

where: *L*_*t*_ varies as 1/*τ*_adh_, the scatter in the steel–bitumen adhesion stress (0.1–0.5 MPa depending on temperature and aging) implies *L*_*t*_ in the range of 150–750 mm for *σ*_rec_ = 300 MPa.

Let us note that this order of magnitude is beyond any practical short-fiber length, regardless of which end of the adhesion stress range is considered. At 60 °C, *τ*_adh_ drops to ≤0.1 MPa and load transfer becomes negligible. Existing studies have proposed techniques for improving adhesion between two different matrices (sandblasting, laser texturing, silane coatings, corrugated fiber profiles), but to date none has been validated in an SMA-mixture composite under thermal activation. Our critical assessment suspects a risk of damage during the heating step, which would alter the interface it was meant to improve.

### 6.2 Anchoring systems

The anchoring system is a mechanical device used to fix reinforcement elements—in our study, this is the Fe-SMA bar—within the bituminous matrix. This reinforcement system primarily ensures effective stress transfer, improves interfacial adhesion, and grants the bituminous matrix a controlled self-healing capacity. Continuous SMA bars or grids require end anchorage to transmit the recovery stress.^63^ Mechanical studs, loop anchors, and bonded grooves all run into the same obstacle: the bituminous pavement has a tensile strength of less than 1 MPa, so that a point anchorage risks pulling out a cone of the surrounding mixture before the SMA stress can be fully mobilized. However, one approach allows the anchorage problem to be entirely circumvented: the use of short dispersed fibers within the matrix (10–50 mm). These integrate well into existing mixing, spreading, and compaction practices. But this approach also has a lock: selective thermal activation of individual fibers is not feasible, and it is risky or even impractical to heat the entire matrix due to its heat sensitivity; consequently, the shape memory effect itself cannot be exploited under these constraints, because short SMA fibers ultimately provide only passive reinforcement, indistinguishable from conventional steel fibers and without any specific SMA advantage.

### 6.3 Durability in a real road environment

Fe-SMAs have become indisputable nowadays, and their performance in concrete/steel (Fig. 4) captivates many researchers and sparks the idea of a technological transposition to bitumen. But to our knowledge, no published study has examined the durability of Fe–Mn–Si in contact with a bituminous mixture. Our critical review reveals several known risk factors, notably: the acidity of aged bitumen (carboxylic acids, ketones), which can attack the metal and promote its corrosion;^59^ in the event of microcracks, chloride ingress through cracks creates pitting sites; initiation of an electrochemical reaction forming galvanic coupling with other metallic components; creep of the matrix pushing the SMA outside its optimal operating range; and thermomechanical fatigue due to daily temperature variations (−10 to +40 °C), not enough to trigger the shape memory effect, but sufficient to gradually degrade the SMA–bitumen interface.^52^

### 6.4 Material cost

Fe–Mn–Si costs 2–5 times more than construction steel and 10–20 times less than NiTi.^2,14^ Typical pavement fibre dosage runs 20–50 kg m^−3^, against an acceptability threshold for road maintenance generally under 5–10 € per m^2^ of extra cost. SMAs are therefore realistic only for critical zones—bridges, viaducts, very high-traffic sections—where reduced intervention frequency and disruption can justify the premium.

### 6.5 Absence of standards and test protocols

No standard, design guide or test protocol currently exists for SMAs in bituminous pavements. Road engineers have no standardised way to characterise crack-closure efficiency in a mixture, measure the recovery stress actually transmitted across the SMA-mixture interface, assess composite durability under temperature cycling, humidity and load, or specify dosage, orientation and anchoring.

### 6.6 Synthesis and prioritization

Table 6 provides an honest and uncompromising, assessment of the technical difficulties hindering the transfer of shape-memory alloys into bituminous mixes. Each bottleneck identified throughout this critical review is classified by order of urgency, whether they involve formulation challenges, rheological hurdles, or uncertainties related to durability. But rather than stopping at this diagnosis, we propose concrete solution pathways for each one, with the aim of turning these obstacles into genuine drivers of innovation. The objective: to offer a clear, operational, and strategic roadmap to guide future research efforts.

**Table 6:** Technological obstacles to integrating SMAs into bituminous mixtures—criticality and solution paths^a^

Obstacle	Criticality	Solution path	Time horizon	Current TRL
Thermal incompatibility (*A*_f_ ≫ 120 °C)	5 (Blocking)	Low-*A*_f_ Fe–Mn–Si alloys (<100 °C)	10+ years	2
Low SMA–bitumen adhesion (<0.5 MPa)	5 (Blocking)	Laser texturing, silane coatings, profiled fibres	5–10 years	3
Anchoring of continuous reinforcements	4 (Major)	High-*T* bonded anchors; dispersed fibres (loses SME benefit)	5–10 years	2
Durability in real environment	5 (Blocking)	Accelerated ageing studies (≥2 years minimum)	3–5 years	1
High material cost	4 (Major)	Reduction of Cr/Ni content; additive manufacturing	5–10 years	2–3
Lack of standardisation	4 (Major)	Participation in RILEM/PIARC working groups	3–5 years	N/A

a Six obstacles block SMA use in bitumen: three critical (thermal incompatibility, weak adhesion, durability) and three major (anchoring, cost, standardisation). Technology remains at TRL 1–3, requiring low-*A*_f_ alloys, better adhesion and standards. Realistic deployment is unlikely before 10–15 years.

### 6.7 Positioning relative to alternative self-healing and repair strategies

The limitations identified in Sections 4 through 6 are specific to thermally activated Fe-SMAs and cannot be generalized to other “smart” or self-healing pavement technologies. Table 7 places the SMA approach alongside three existing approaches that have been successfully applied and have a more mature track record directly in bituminous mixtures. Through this table, we position the present findings within the broader landscape of self-healing pavements, rather than assessing the technology in isolation.

**Table 7:** Benchmarking the Fe–Mn–Si SMA route against alternative self-healing/active-repair strategies for bituminous mixtures

Technology	Activation trigger	Reported performance range	TRL (bituminous-mixture context)	Key limitation
Fe–Mn–Si SMA (this review)	External heating to *A*_f_ (120–260 °C)	80–90% crack closure, +50–70% shear/fatigue gain—concrete/steel only; no bituminous-mixture data^3,18,55^	1–2	Activation temperature destroys the host matrix before transformation completes (Section 4)
Induction heating of conductive steel/aluminium fibres	Electromagnetic induction; bitumen softens locally at 60–90 °C, below degradation onset	Stiffness/fatigue-life recovery roughly 60–90% per healing cycle across independent studies, decreasing with repeated cycles^24,26^	6–7 (Field trials reported)	Repeatable but with diminishing returns; energy input and fibre-dispersion uniformity; effective mainly at microcrack scale
Microencapsulated rejuvenators	Mechanical crack propagation ruptures capsules; ambient temperature	Strength/stiffness recovery typically in the tens-of-percent range in laboratory specimens^26^	3–4	Single-use unless multi-walled/reactive capsules are used; capsule survival during hot mixing (150–180 °C) is unresolved
Bio-based/polymer-modified rejuvenating binders	Diffusion of rejuvenator through the binder film; ambient to moderate temperature	Partial restoration of fatigue life and low-temperature ductility over weeks-to-months timescales^30^	4–5	Repeatable and cumulative but slow; incompatible with rapid reopening of high-traffic roads

Table 7 well summarizes what this critical review demonstrates: the commissioning of self-healing bituminous pavements with the help of AMF could be possible in synergy. Nowadays, induction-heated fibers and encapsulated rejuvenators already reach TRL 4 to 7, with solid laboratory evidence on asphalt mixtures. However, this evidence comes from a small number of teams and setups, with no field testing, and “recovery” is not measured in the same way across authors (stiffness, cracking, fatigue … all of these do not indicate the same thing). On the other hand, the thermally activated Fe–Mn–Si route is too immature to be applied to bitumen at present, particularly from its very first mechanistic step (Section 4). Extrapolating the good results obtained on concrete or steel (Fig. 4) to justify a technological transposition to bitumen would be a serious scientific error. Because here, the problem is not simply a delay or a lack of calibration: it is that the most elementary step—reaching *A*_f_ without destroying the bituminous matrix—has simply never been achieved. A far more fundamental failure (Table 8).

**Table 8:** TRL scale for SMA-mixture application in road infrastructure^a^

TRL	Definition	Example for SMA-mixture
1	Basic principle observed	Shape memory effect demonstrated on Fe–Mn–Si in the laboratory
2	Technology concept formulated	Proposal to use Fe–Mn–Si to close cracks in mixtures
3	Experimental proof of concept (specimens <10 cm)	Tensile test on an SMA-mixture specimen; measurement of transmitted *σ*_rec_
4	Laboratory validation (specimens >30 cm)	Crack-closure test on a 30 × 30 cm mixture slab
5	Validation in relevant environment	Cyclic fatigue test on a 1 × 1 m slab with integrated SMA activation
6	Demonstration in real environment (<100 m pilot)	Installation and activation of Fe-SMA on a 50 m service lane
7	Demonstration in operational environment (>500 m, real traffic)	Application on a hard shoulder or low-traffic lane
8	System qualified and certified (standards available)	PIARC technical guide; standardised activation procedure
9	Large-scale commercial deployment	Routine use by road maintenance agencies

a SMA–bitumen systems currently sit at TRL 1–3 (basic research, small specimens). Three blocking obstacles—thermal incompatibility, weak adhesion, durability—must be solved to reach TRL 4–5. Realistic deployment beyond TRL 8 requires 15–20 years and remains highly uncertain.

### 7.3 Scenario analysis 2026–2035

This review proposes three possible probabilistic scenarios in the evolution of this innovative AMF pathway, inspired by Thomas Edison who said: “every result of a scientific study – success or failure – modifies the probability of achieving an innovation”. We also propose the attitude to adopt for each scenario:

• Optimistic scenario (probability <10%): when we succeed in preparing an alloy with a low *A*_f_ (<100 °C, *σ*_rec_ > 200 MPa) reaching maturity by 2032; the localized induction heating technique is validated from 2033; 500 m pilot sections are built in 2034–2035; limited commercial deployment (bridges, viaducts) follows around 2038–2040. The approach would be qualified as successful.

• Median scenario (probability ≈50%): when we succeed in developing a Fe-AMF with an *A*_f_ that reaches 110–120 °C rather than 100 °C; ultra-short heating pulses keep bitumen degradation below 0.5 mm; *τ*_adh_ only reaches 1–1.5 MPa, which is sufficient for localized spot repairs (pavement joints, bridge approach slabs) but nothing more widespread; we could project the first operational applications around 2040–2045. The technology would be said to be on the right track or qualified as promising (Table 9).

**Table 9:** Research axes, target TRL, objectives, and go/no-go criteria^a^

Axis	Target TRL	Objective	Go/no-go criterion (decision date)
1	TRL 4	Develop Fe–Mn–Si with *A*_f_ < 100 °C (ideally 80 °C) and *σ*_rec_ > 200 MPa	If *A*_f_ < 100 °C and *σ*_rec_ > 150 MPa are not achieved by 2030, shift to ultra-rapid localised heating (axis 2)
2	TRL 4	Demonstrate localised Joule/induction heating keeping bitumen below 100 °C at 1 mm from the SMA	If bitumen exceeds 120 °C for >10 s under any protocol with *σ*_rec_ > 200 MPa, abandon thermal activation by 2032
3	TRL 4	Achieve *τ*_adh_ > 2 MPa at 20 °C and >0.5 MPa at 60 °C on the SMA–mixture interface	If *τ*_adh_ < 1 MPa after ageing, switch to mechanical anchoring (profiled fibres, non-circular geometries)
4	TRL 3	Assess SMA–mixture durability under freeze–thaw, chlorides, UV and cyclic loads (2-year equivalent)	If *σ*_rec_ drops >50% after ageing equivalent, durability is insufficient for road applications
5	N/A	Produce standardised test protocols and a design guide (dosage, orientation, anchoring, activation)	Submit protocol proposal to RILEM TC or PIARC by 2030

a A five-axis roadmap targets TRL 4: low-*A*_f_ Fe–Mn–Si alloys (*σ*_rec_ > 200 MPa), localised heating (<100 °C), adhesion (*τ*_adh_ > 2 MPa), 2-year durability, and standardisation by 2030. Strict go/no-go gates mean failure in any axis redirects or abandons the strategy.

• Pessimistic scenario (probability ≈40%): when no viable low-*A*_f_ Fe-AMF alloy emerges by 2032, the continuous heating technique inevitably degrades the bitumen, and durability remains insufficient (loss of *σ*_rec_ greater than 70% after one equivalent year). We would trigger a technological abandonment process. That is to say, if no Fe–Mn–Si alloy reaches *A*_f_ < 120 °C with *σ*_rec_ > 150 MPa after ten thermal cycles, or if the bitumen within a 1 mm radius of the AMF exceeds 120 °C for more than 5 s under an activation protocol, the AMF-asphalt pathway should be abandoned and we will redirect effort towards encapsulated rejuvenators, induction heating of conventional steel fibers, polymer-modified binders with molecular healing capacity, or bio-based rejuvenators.

## 8 Conclusion

This review set out to answer a precise question: can thermally activated Fe–Mn–Si SMAs close cracks in a bituminous mixture without degrading the bitumen? After examining the thermomechanical behavior of Fe–Mn–Si, bitumen rheology, proposed self-healing mechanisms, and the technological obstacles blocking their path, our answer to this question with current materials leaves little doubt: it is no, and extrapolating this technology from data taken from studies on concrete will not change that. We identify three absolute barriers that currently seem to block this innovation:

• Thermal incompatibility: Fe–Mn–Si requires 160–260 °C for its activation, but bitumen irreversibly degrades above 120 °C, which explains a thermal conflict; and even the use of localized heating brings the bitumen beyond 120 °C in less than ten seconds.

• Viscoelastic stress dissipation: the bituminous mixture creeps under *σ*_rec_, causing the recovery stress to drop from about 200 MPa to less than 50 MPa within a few days, without guaranteeing crack closure.

• Insufficient interfacial adhesion: the value of *τ*_adh_ is equal to or less than 0.5 MPa, which limits the effective anchorage length to less than 12.5 mm per 100 MPa of recovery stress, far below what practical fiber lengths allow.

Note that the figures of 80–90% crack closure and +50–70% fatigue life extension so often touted in the concrete/steel literature simply cannot apply to bituminous mixtures at the present time. This is not a failure of the SMA concept, but it is what happens when a concept is extrapolated without being adapted.

To hope to apply this innovation in the coming years, three research priorities emerge with concrete quantitative objectives:

• Priority 1—preparation of low-*A*_f_ Fe-SMA: develop Fe–Mn–Si with *A*_f_ below 100 °C (ideally 80 °C) and *σ*_rec_ above 200 MPa.

• Priority 2—mastery of ultra-rapid localized heating technique: optimize Joule or induction pulses of less than 5 s that activate the SMA without bringing the surrounding bitumen beyond 100 °C.

• Priority 3—dedicated characterization and improvement of the SMA–bitumen interface: build standardized pull-out and shear tests for SMA–bitumen composites, targeting *τ*_adh_ above 2 MPa at 20 °C and above 0.5 MPa at 60 °C.

In view of the structure of these priority axes, if no significant progress toward these objectives materializes by 2030–2032, the field should conclude that SMAs are not suitable for bituminous pavements and redirect efforts toward alternative self-healing strategies, such as encapsulated rejuvenators, conventional induction-heated fibers, bio-based healing agents.

Establishing that a pathway is not viable is a legitimate scientific result in itself, not a failure to be reported. Shape memory alloys remain truly valuable materials, with a solid track record in concrete, steel, and aerospace applications. But a bituminous pavement is not a small concrete bridge: it is viscoelastic, temperature-sensitive, prone to creep, and chemically demanding in ways that concrete simply is not. Carrying a technology across this border without adapting it is not innovation—it is optimism disguised as progress. What this field needs now is honest, rigorous, and tailored research. The road to truly smart pavements is long, but possible, and it will not be paved by extrapolation alone.

## Conflicts of interest

The author affirms that this research was conducted in the absence of any commercial or financial relationships that could be construed as a potential conflict of interest.

## Data Availability

All data analysed in this review are available from the corresponding author upon reasonable request.
